# Breast Cancer Prevention-Is there a Future for Sulforaphane and Its Analogs?

**DOI:** 10.3390/nu12061559

**Published:** 2020-05-27

**Authors:** Dominika Kuran, Anna Pogorzelska, Katarzyna Wiktorska

**Affiliations:** 1 Department of Pharmacology, National Medicines Institute, 00-725 Warsaw, Poland; d.rogulska@nil.gov.pl; 2 Department of Drug Biotechnology and Bioinformatics, National Medicines Institute, 00-725 Warsaw, Poland; a.pogorzelska@nil.gov.pl; 3 OncoBoost Ltd., 02-089 Warsaw, Poland

**Keywords:** sulforaphane, isothiocyanates, breast cancer, chemoprevention, anticancer activity

## Abstract

Breast cancer is the most prevalent type of cancer among women worldwide. There are several recommended methods of breast cancer prevention, including chemoprevention. There are several approved drugs used to prevent breast cancer occurrence or recurrence and metastasizing. There are also a number of new substances undergoing clinical trials and at the stage of initial study. Studies suggest that dietary factors play a crucial role in breast cancer etiology. Epidemiological studies indicate that in particular vegetables from the *Brassicaceae* family are a rich source of chemopreventive substances, with sulforaphane (SFN) being one of the most widely studied and characterized. This review discusses potential applicability of SFN in breast cancer chemoprevention. A comprehensive review of the literature on the impact of SFN on molecular signalling pathways in breast cancer and breast untransformed cells is presented. The presented results of in vitro and in vivo studies show that this molecule has a potential to act as a preventive molecule either to prevent disease development or recurrence and metastasizing, and as a compound protecting normal cells against the toxic effects of cytostatics. Finally, the still scanty attempts to develop an improved analog are also presented and discussed.

## 1. Introduction

According to the latest global cancer statistics database (GLOBOCAN) released in September 2018, in the 21st century cancer will be the main cause of death and the single most important barrier to increasing life expectancy in every country of the world. Among cancers, breast cancer is the second most commonly diagnosed cancer in both sexes worldwide and the most prevalent cancer in women in almost all of the countries worldwide. 2.1 million newly diagnosed female breast cancer cases in 2018, accounting for almost 1 in 4 cancer cases among women were expected. Although it has a relatively low fatality rate due to frequency of occurrence, it still has the highest mortality rate of any cancer in women [1].

Studies suggest that dietary factors play a crucial role in breast cancer etiology [2]. Epidemiological studies show that a diet rich in fruit and vegetables significantly reduces the risk of developing lung, esophageal, laryngeal, pancreatic, colorectal, gastric and prostate cancers, as well as breast cancer [3,4,5]. Vegetables from the *Brassicaceae* family are a rich source of chemopreventive substances. These include 350 plant species, such as cabbage, broccoli, cauliflower and Brussels sprouts [6,7,8]. It has been shown that phytochemical compounds and their precursors contained in these vegetables reduce the risk of developing cancer, including breast cancer [9,10]. The anti-cancer properties of these plants are due to the glucosinolates (GLS) they contain, which, due to the activity of the enzyme myrosinase, undergo enzymatic degradation to isothiocyanates (ITC) [11]. The most commonly studied ITC is sulforaphane (SFN), which shows the potential to inhibit and reverse the process of carcinogenesis, and to destroy cancer cells by apoptosis. Now, the literature offers an increasing number of papers on natural and synthetic SFN analogs and derivatives with their biological activity significantly higher than that of SFN. This paper reviews and discusses the activity of this group of compounds in relation to breast cancer, which seems to be their specific target.

## 2. Epidemiology and Characteristics of Breast Cancer

After lung cancer, breast cancer is the second most commonly diagnosed cancer in both sexes combined and accounts for 11.6% of all cancer cases. The disease is the most frequently diagnosed cancer in the vast majority of the countries (154 of 185), as well as the leading cause of cancer death in more than half of the countries (100 of 185 countries) [12]. Moreover, due to risk factors, the incidence and mortality of breast cancer is five times higher in Western countries than in Asian ones. Also importantly, breast cancer onset takes place relatively earlier in age compared to other major cancers [13].

Breast cancer may be hereditary. *BRCA1* (*breast cancer susceptibility gene 1*) is the first cancer susceptibility gene discovered. Inherited mutations in the *BRCA1* gene are behind an increase in the breast and ovarian cancer risk in women. It is estimated that about 5% of all breast cancer cases are associated with *BRCA1* mutations. This gene encodes a protein that has been implicated in multiple nuclear functions, such as transcription, cell-cycle regulation and DNA repair [14,15,16]. While BRCA1 protein is crucial for cells, it plays a central role in cell maintenance, growth, cell cycle and apoptosis. Its alteration or loss could therefore lead to an uncontrolled cell proliferation (carcinogenesis). The BRCA1 alteration or loss can occur as a result of exposure of the body to carcinogens, such as polycyclic aromatic hydrocarbons (PAH), heavy metals or peroxides [15].

Hereditary and genetic factors, including a personal or family history of breast or ovarian cancer as well as inherited mutations (in *BRCA1*, *BRCA2*, and other breast cancer susceptibility genes) account for 5% to 10% of breast cancer cases. Studies of migrants have shown that most of breast cancer are a sporadic and non-hereditary factors (i.e., due to gene mutation that occurred by chance) are the major driver of the observed international and inter-ethnic differences in breast cancer incidence [17]. The detection of the estrogen receptor (ER), progesterone receptor (PR) and human epidermal growth factor receptor (HER2) is crucial for prognostic evaluation and treatment choice of breast cancer for clinical practice. The ER/PR status is essential for clinical and therapeutic care provided to breast cancer patients [16,17]. This classification helps to choose hormonal treatment for ER/PgR-positive patients and chemotherapy for the ER/PgR-negative patients. Incidence of breast cancer increases with age, with elderly patients being more responsive to hormone therapy if they are ERα or PR-positive. Generally, more than 70% of women with breast cancer are ER-positive, while those PgR-positive represent 50%. ERα-negative breast cancer has a poor prognosis partly due to the lack of target-directed approaches. The most devastating disease with high morbidity and mortality rate is triple negative breast cancer (TNBC). TNBC is defined by the absence of the three most commonly targeted receptors: ER, PR and HER2. Patients with TNBC develop larger tumor mass, and the disease progression is more aggressive [18,19,20,21,22,23].

Breast cancer is a complex, molecularly, pathologically, and epidemiologically heterogeneous disease. The solid breast tumors at majority comprise of differentiated cells that have limited self-renewal abilities. Cancer stem cells (CSCs) is a small subpopulation of cells within a tumor capable of self-renewal, differentiation and able to undergo epithelial-to-mesenchymal transition (EMT), a known mechanism in cancer metastasis. The CSC model of tumor growth explain the intra-tumor heterogeneity. It was beginning of 21th century when it was proposed that mammary stem cells can undergo transformation due to genetic mutations [24,25]. Overall preclinical evidence suggests that many anti-cancer agents such as sunitinib, doxorubicin and gemcitabine only inhibit the growth of differentiated cancer cells, without eliminating or possibly even expanding CSC populations [15,16,26]. Hence, many drug discovery research has focused on breast CSCs as a new breast cancer therapy target. Only recently, a comprehensive review was published which discussed the crucial role of breast CSCs in breast cancer development and recurrence and novel therapies being explored [27].

Factors increasing the risk of breast cancer primarily include lack of physical activity, high body mass index, especially in the postmenopausal period, and a diet poor in unsaturated fats, vegetables and fruit. A comprehensive diet rich in fruit and vegetables can reduce the risk of breast cancer by up to 30% [28]. The epidemiological studies and additional evidence derived from studies with compounds modulating the estrogen or the progesterone receptors have also shown that the length of exposure to endogenous hormones, as determined by an early menarche or a late menopause, is a risk factor for breast cancer [29].

## 3. Breast Cancer Prevention

Despite progress in diagnosing breast cancer in women, the mortality rate of this disease remains very high. This is due to the fact that in many women the breast cancer is detected too late, so that the chances of survival are much lower than in case of women in whom it was detected at an early stage. The basic preventive methods include breast self-examination, mammography and ultrasound [30,31].

The aim of breast self-examination for women is to learn the breast topography, know how their breasts normally feel and be able to identify changes in the breast should they occur in the future. Breast self-examination should be used in combination with mammography and clinical breast examination rather than as a substitute for either method. In fact, it is currently debated whether breast self-examination alone can reduce the number of cancer deaths [32,33].

Mammography is another screening tool used for detecting early breast cancer. The objective of population-based mammography screening is to reduce mortality and morbidity from breast cancer through early detection and treatment of malignancies. There is sufficient evidence for the efficacy of screening women aged 40 to 70 by mammography in reducing mortality from breast cancer [33]. However, mammography is an imperfect and limited test, especially due to dense breast tissue. Dense breast tissue may mask an underlying tumor and therefore decreases mammographic sensitivity. Moreover, dense breast tissue is an independent risk factor for breast cancer. Compared to women with predominantly fatty breast tissue, the risk of women with dense breast tissue of developing breast cancer is 4 to 6 times higher [34].

Ultrasound examination of the breast is a popular choice, since it is well-tolerated by patients, widely available, does not require intravenous contrast or ionizing radiation, and is relatively inexpensive. However, the ultrasound examination of the breast is also an imperfect test, associated with low specificity compared to mammography, and it typically requires a highly experienced technologist or physician to perform an examination. However, the ultrasonographic method may show small, node-negative breast cancers not seen in mammography. Combining single screening ultrasonographic examination with mammography for women at elevated risk of breast cancer allows for an increased detection rate of breast cancers that are predominantly small and node-negative [34].

Women with a *BRCA1* and *BRCA2* mutation and other women with a family history of breast cancer risk may choose to undergo bilateral prophylactic mastectomy. Mastectomy reduces breast cancer risk by 90% to 100% after 3–13 years of follow-up [35,36].

### 3.1. Chemoprevention

Apart from the methods of breast cancer prevention described above, chemoprevention is also used. Cancer chemoprevention is defined as the use of natural, synthetic, or biochemical agents to reverse, suppress or prevent carcinogenic process resulting in a neoplastic disease [37]. The idea of chemoprevention emerged in the late 1960s, created by Wattenberg and Sporn. In animal models, they observed that certain food-derived compounds may inhibit carcinogenesis. Their findings gave rise to the development of chemoprevention [38,39]. Since carcinogenesis has multiple stages, there are three chemoprevention types identified, including the following:

Primary chemoprevention, the so-called anti-initiation chemoprevention, which is a strategy involving healthy people who, however, constitute a high risk population, genetically predisposed to developing a particular type of cancer. This type of chemoprevention is based on the use of cancer inhibitors at the initial stage [40,41].

Secondary chemoprevention is described as an anti-promotion or anti-progression strategy. It inhibits tumor progression and serves as a therapy model for early detection of pre-cancerous conditions [40,41].

Tertiary chemoprevention is designed to prevent the relapse of the disease and the formation of another primary tumor/disease [40,41].

Chemoprevention agents are divided into two groups. The first one is a group of blocking agents, the so-called anti-initiation agents. The agents belonging to this group may inhibit the metabolic activation of the carcinogen, block the interaction of the carcinogen with the connective tissue by increasing detoxification intensity, increase the effectiveness and efficiency of DNA repair systems. Blocking agents can also inactivate reactive metabolites and free radicals to prevent their reaction with DNA and induce apoptosis. The other group of agents is referred to as suppressive or anti-progressive, and it inhibits the process of carcinogenesis by inhibiting inflammatory processes, modifying signal transduction pathways, removing reactive oxygen species and promoting cancer cell apoptosis [41,42].

#### 3.1.1. Registered Drugs and Clinical Trials in Breast Cancer Prevention

Currently, only two drugs used in breast cancer prevention have been granted the marketing authorization, namely tamoxifen and raloxifene. Both of them are selective estrogen receptor modulators (SERM) and were shown to act as estrogen antagonists in breast tissue [29,43].

Tamoxifen has been shown to decrease the risk of breast cancer in both pre- and post-menopausal women. Tamoxifen (Nolvadex, Tamizam, Tomoplex) was approved in the United States (U.S). by the Food and Drug Administration (FDA) in 1998 as a drug for the prevention of breast cancer in women at high risk, while in the European Union (EU) it was approved in 2016 [44]. Clinical trials have confirmed the efficacy of tamoxifen, which inhibited the development of breast cancer in the other breast in women with a history of breast cancer. However, the use of tamoxifen presents drawbacks such as thromboembolic complications and the risk of endometrial cancer [43].

Raloxifene (Evista, Raloxifene), which was initially approved in 1997 for osteoporosis treatment and prevention, was approved by the FDA in 2007 for breast cancer risk reduction in postmenopausal women who either had osteoporosis or were at high risk for invasive breast cancer [45]. In 2010, raloxifene was approved for medical use in the UE, but only for treatment and prevention of osteoporosis in postmenopausal women [44].

The other compounds are undergoing clinical trials. The ClinicalTrials.gov database shows an increasing number of studies dedicated to the development and research of new oncological drugs and it is estimated that breast cancer research accounted for 28% of all of such studies [46]. The clinical studies described herein that are designed to select effective drugs for breast cancer chemoprevention have been conducted over the last decade. They primarily include drugs registered for other indications. The results published so far have suggested few promising structures (Table 1) with chemopreventive properties superior to those of the registered tamoxifen and raloxifene [47,48,49,50,51,52,53,54,55,56,57].

One of the best candidates is Anastrozole (Arimidex), an aromatase inhibitor that blocks estrogen production, approved by FDA and EMA (European Medicines Agency) to treat breast cancer in postmenopausal women (Table 1). The papers published during Phase 2 clinical trials have shown that anastrozole effectively reduced the incidence of breast cancer in high-risk postmenopausal women, however it had a poorer impact on estrogen-receptor-negative cancers [58]. Recently, the results of an over-5 year treatment were published, showing a significant efficacy of anastrozole in preventing breast cancer (both invasive and ductal carcinoma in situ) in the post-treatment period [59]. Another aromatase inhibitor used to treat breast cancer, exemestane (Aromasin), was shown to reduce breast cancer risk with similar efficacy in high-risk postmenopausal women [48]. Anastrozole and exemestane were shown to have greater efficacy than tamoxifen and raloxifene as well as reduced side effect profile [47,58,60].

Lasofoxifene (Fablyn)–a nonsteroidal SERM undergoes tests under Phase II clinical trial is approved for prevention and treatment of osteoporosis (Table 1). Preclinical laboratory evidence showed that Lasofoxifene prevented experimentally induced breast cancers without inducing endometrial hyperplasia. The clinical trial results were in line with the in vitro study results. A 79% reduction in the rates of invasive breast cancer and an 83% reduction in ER-positive breast cancer in women who received Lasofoxifene was reported. Moreover, the drug showed significantly higher efficacy than the related SERMs tamoxifen and raloxifene [49,61].

Pasireotide (Signifor), a somatostatin analog which was approved by the FDA in 2012 as an orphan drug for the treatment of Cushing’s disease inhibits the activity of insulin-like growth factor-1 (IGF-1), which plays a central role in the mammary development and carcinogenesis (Table 1). The results of Phase 1 proof of principle trial have shown that inhibition of the IGF-1 with Pasireotide decreases cell proliferation and increases apoptosis in precancerous breast lesions, which indicates that Pasireotide could be an effective alternative for breast cancer chemoprevention [50,62,63].

Regrettably, only few compounds prove successful in clinical trials. Arzoxifene, an initially promising SERM, failed in Phase 3 clinical trial. Substantial side effects were reported, and given the overall results, Arzoxifene was not considered a potential candidate for clinical development (Table 1) [51]. Likewise, for lovastatin (Mevacor), a statin used to prevent and treat coronary heart disease, no significant breast cancer biomarker modulation was reported after a 6-month course of oral intake in women with a high hereditary breast cancer risk (Table 1). Also, there was neither beneficial or adverse effect reported after 12 months of randomized, double-blinded placebo-controlled intervention study on soy isoflavones supplementation in treating women at high risk [52,64].

ClinicalTrials database also includes several studies, mainly Phase 2 trials, which either are still underway or there are no results available yet from them. At present, it is therefore impossible to determine whether the structures tested will prove promising in terms of both chemoprevention efficiency and potential side effects. To provide a full picture, they will be briefly discussed below.

Alendronate, a bisphosphonate (BP) drug for treatment of osteoporosis, is in Phase 1 clinical trial. The study is designed to determine the effect of the drug on the levels of T-cells (*γ δ*) and on the levels of breast cancer epithelial cells in women at high risk due to *BRCA* gene mutation, or a persistent family history of breast cancer who are to undergo preventive mastectomy. The study is still underway; it is to be completed in March 2020 [53].

Letrozole, a nonsteroidal aromatase inhibitor used in hormone therapy for breast cancer, is in Phase 2 clinical trial (Table 1). The object of the trial is to assess the level of K*i*-67 proliferation marker in breast epithelial cells and side effects, i.e., hot flashes in postmenopausal women from the so-called high risk group who have previously undergone hormone replacement therapy. The trial was completed in October 2019; its results, however, have not yet been published [54].

Another Phase 2 preventive study is being carried out on deslorelin efficacy in combination with estradiol and testosterone in *BRCA* gene mutation carriers. Deslorelin is a gonadotropin-releasing hormone agonist (GnRHA) used in veterinary medicine for various indications, e.g., induction of ovulation (Table 1). The object of the study is to evaluate the correlation of changes in mammographic and magnetic resonance imaging (MRI) densities with breast tissue morphometrics and biomarkers as well as prospects for risk reduction options and the impact on quality-of-life indices. This study was completed in September 2019, but no results have been published yet [55].

Yet another Phase 2 clinical trial relates to a combination of sulindac with adjuvant hormonal therapy of early stage ER+ breast cancer. Sulindac is a nonsteroidal anti-inflammatory drug (NSAID) used in the treatment of inflammatory conditions (Table 1). The objects of the study include an assessment of chemopreventive potential of sulindac to decrease breast density and reduce breast cancer development risk. This study was completed in June 2019, but no results have been published yet [56].

Metformin hydrochloride (Glucophage), the first-line medication for the treatment of type 2 diabetes, is in Phase 3 clinical trial (Table 1). The object of the trial is to evaluate the presence or disappearance of cytological atypias and the level of biomarkers characteristic for breast cancer in healthy women with *BRCA1* or *BRCA2* gene mutation or in premenopausal women. The study is to be completed in June 2022 [57].

#### 3.1.2. Natural Compounds in Breast Cancer Prevention: Preclinical Studies

Natural products derived from a variety of sources can be very useful in the prevention of occurrence and development of cancer, including breast cancer. Various compounds were suggested at the preclinical stage to have great efficacy. The research of a number of those, such as curcumin, genistein, resveratrol, epigallocatechin gallate (EGCG) (Table 2), is still in progress [65,66,67,68,69].

Curcumin (diferuloylmethane), a naturally occurring phytochemical, is responsible for the yellow color of the commonly used spice turmeric (Curcuma longa Linn.), (Table 2). Curcumin has a widespread medical activity including that against breast cancer. Choudhuri et al. showed that it can induce apoptosis by increasing p53 levels, which in turn induces Bax expression in MCF-7 cell line [66]. Curcumin inhibited in a dose-dependent manner the proliferation of MCF-7 cells and decreased the expression of urokinase-type plasminogen activator (uPA) and NF-κB binding activity [70].

Genistein is an isoflavonoid present in soy products, which has been proposed as the agent responsible for lowering the breast cancer rates in Asian women (Table 2) [71]. It was shown that in MDA-MB-231 cells treated with genistein an up-regulation of Bax and p21WAF1 expression as well as down-regulation of Bcl-2 and p53 expression occurs and apoptosis is induced via a p53-independent pathway [67].

Resveratrol (trans-3, 5, 4′-trihydroxystilbene) is a phytoalexin present in grapes and red wine (Table 2). In MCF-7 breast cancer cell line, the use of resveratrol induced an apoptotic effect and inhibited cell growth via the caspase-9 Akt pathway [72]. Other studies found that resveratrol inhibited NF-*κ*B, a regulator of Bcl-2 expression as well as calpain protease activity in MCF-7 breast tumor cells and induced apoptosis by interfering with the estrogen receptor (ER*α*)-dependent PI3K pathway [68].

EGCG is the most abundant catechin found in green tea. EGCG (10–50 µm) was found to suppress, via ER, the estrogenic, proliferation-stimulating activity of polychlorinated biphenyl PCB 102 in estrogen-sensitive breast cancer cell line MCF-7/BOS. PCB 102 is an environmental carcinogen and may stimulate the growth of estrogen-sensitive tumors, including breast tumors. Hence, the inhibition of its estrogenic activity may be beneficial in breast cancer chemoprevention (Table 2) [69].

According to literature data, SFN–a product of enzymatic reduction of glucoraphanin present in a Brassica vegetable is a structure with emerging chemopreventive potency. A rapid rise in the number of publications on SFN and breast cancer has been observed in the last years. In recent years, there were published annually c.a. 200 SFN-related publications, with almost 10% there of regarding breast cancer, according to PubMed database.

## 4. SFN: Structure, Occurrence and Metabolism

**Figure 1:**
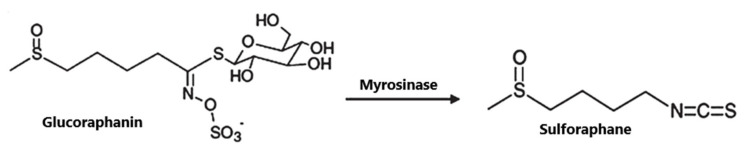
Schematic diagram of the formation of sulforaphane (SFN) (based on [74] modified).

SFN (1-isothiocyanate-4-methyl-sulfinylbutane) is the best known compound of the ITC group, Figure 1. SFN is a light yellow oily liquid with a molar mass of 177 g/mol. SFN is obtainable by way of chemical synthesis. The products of this reaction depend on pH, temperature and ions Fe^3+^. The highest reaction yield is obtained at the temperature of 60 °C and pH 5.0 to 7.0 [73].

The compound was first synthesized in 1948, while in 1958 it was isolated from the leaves of hoary cress *(Lepidium draba).* Then, in 1992, it was found that plants from the *Brassicaceae* family owe their chemopreventive properties to SFN [73,75,76].

SFN is generated during the destruction of plant tissue as a result of enzymatic hydrolysis of the precursor glucoraphanin contained in plants, (Figure 1), and its bioavailability depends on the yield of that reaction. Glucoraphanin (GFN) is found in vegetables in variable amounts, depending on the type of vegetable cultivar grown and on the time after the germination of the plant. GFN is most abundant in broccoli, with dry matter containing 0.8 to 21.7 µmol/g. It was shown that other plants of the *Brassicaceae* family, such as cabbage, Brussels sprouts, kohlrabi, radish and turnip are also rich in SFN precursor [74,77].

The myrosinase enzyme hydrolyzes GFN to SFN (Figure 1), however GFN is also degraded by gut microbiota and absorbed in the colon which is a major route of SFN formation in humans in case of thermal degradation of myrosinase (in cooked vegetables), however the efficiency of this route is low. According to data, consumption of broccoli resulted in higher blood and urine levels of SFN and its metabolites when broccoli was raw instead of cooked; when broccoli was eaten raw, bioavailability of 37% was reported, versus bioavailability of 3.4% when cooked broccoli was consumed. A SFN peak in plasma were observed 1.6 h and 6 h after consumption of raw and cooked broccoli, respectively [78].

Glutathione (GSH) conjugation is a major route for elimination of ITC in mammals. GSH conjugation is catalyzed by glutathione S-transferase (GST), which lowers the pKa value of GSH cysteine residues, so that the latter is present as a thiolate anion in physiological conditions, which enhances its nucleophilic properties to attach to other electrophiles, including the central carbon of SFN. After conjugation, SFN-GSH is enzymatically degraded to SFN-cysteine-glycine. Then, via enterohepatic circulation, they enter the liver and are acetylated to N-acetyl derivatives (SFN-N-acetylcysteine) or mercapturic acid derivatives. The acetylation products are then transported to the kidneys and excreted in the urine [79,80,81]. According to the literature data, 1 h after oral administration of 200 µmol/L broccoli sprouts, the level of isothiocyanates in plasma, serum and erythrocytes was 0.94–2 µmol/L, and the half-life was 1.8 h [82].

Due to short half-life, the bioavailability and efficacy of SFN may be low. Hence, some effort are made to improve stability of SFN in body fluids. One approach is to use nanaocarriers based on either natural or the synthetic polymers [83,84]. Soni et al. have described preparation of SFN-loaded nanostructured lipid carriers. It was shown that the release of SFN was higher than in comparison to SFN suspension, as well as gut permeation what resulted in a 5-fold increase in relative oral bioavailability in rats [84]. In turn loading SFN into PCL-PEG-PCL micelles led to elongation of the circulation time and to increase in the therapeutic efficacy of SFN. It was shown that in comparison with the free SFN, the SFN-loaded micelles residence time increased from 0.5 to 4 h and the area under the concentration-time curve up to 50 folds [85]. These results are encouraging and indicate that the loading SFN in the appropriate nanocarrier is a method for the effective improvement of SFN efficacy due to its better stability, longer circulation time and eventually increased bioavailability.

## 5. Role of SFN in Breast Cancer Prevention

**Figure 2:**
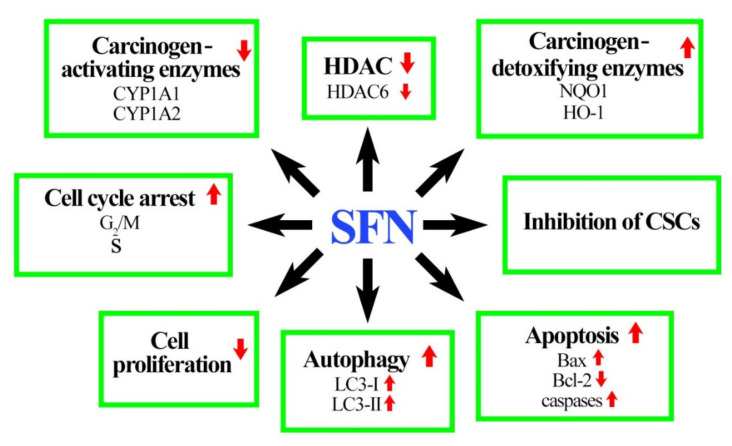
Mechanisms of SFN activity in breast cancer tissue.

As regards breast cancer chemoprevention, mechanism of action of SFN is multidirectional and includes e.g., increased levels of detoxification enzymes, decreased enzymatic activity of P450 cytochrome (CYP1A1, CYP1A2), reduced cancer cell viability/proliferation through inhibition of cell cycle, induction of apoptosis and autophagy as well as elimination of cancer stem cells (CSCs). The effect of SFN on these molecular targets, presented in Figure 2, is discussed in detail below.

### 5.1. In Vitro Studies Demonstate the Efficacy of SFN

Literature on SFN used in breast cancer mostly includes in vitro studies using cell lines derived from patients representing different types of the cancer, different stages of the disease and different tissue types. These lines differ in terms of molecular structure and sensitivity to chemotherapeutic agents. The most commonly used cell lines representing cancer and normal mammary tissue in in vitro studies are summarized in the Table 3.

#### 5.1.1. Phase I of Xenobiotic Metabolism

The first phase of xenobiotic metabolism involves the reactions of hydrolysis, oxidation and reduction which result in the formation of polar functional groups. These reactions yield products that are more hydrophilic than substrates, which facilitates their excretion [86]. Phase I xenobiotic metabolism reactions are usually catalyzed by the cytochrome P450 enzyme system (CYP450). The primary function of these enzymes involves the catalysis of the reaction where one atom of the oxygen molecule is introduced into the substrate molecule, resulting in a hydroxyl derivative. Then, the other atom from the oxygen molecule is reduced to a water molecule [87].

CYP450 isoenzymes (e.g., CYP1A and CYP1B) catalyze reactions where pro-cancerogenic compounds (such as polycyclic aromatic hydrocarbons, PAHs) are metabolically activated to active carcinogens capable of forming adducts with intracellular molecules, such as DNA, giving rise to mutations, followed by activation of oncogenes and inactivation of suppressor genes [88]. The inhibition of these enzymes is considered to be the key mechanism of chemoprevention of carcinogenesis, in particular chemically induced carcinogenesis.

The study performed by our team has shown that SFN may reduce the induction of CYP450 expression due to PAH exposure in MCF-7 cell line. We have shown that 0.5 µm, 1 µm and 2.5 µm doses of SFN inhibit the activity of CYP1A1 and CYP1A2 induced by: nontumorigenic anthracene and highly tumorigenic dibenz[a,h]anthracene. Inhibition of CYP1A2 versus CYP1A1 by SFN was far more potent. The observed activity drop was linked to the protein level decrease, resulting from the inhibition of AhR receptor translocation into the nucleus, which in turn is a trigger of AhR-dependent enzymatic protein production, including CYP1A1 and CYP1A2 [89].

In general, the data on the effect of SFN on CYP450 enzymatic activity is insufficient and inconsistent, and they vary between cell lines and enzyme isoform types. For example, the study by Licznerska et al. showed an increase in transcript level of CYP1A2 in the non-tumorigenic MCF-10A cell line upon SFN stimulation (5 µm, 10 µm). The very same study, however, showed that 5 µm SFN slightly reduced the expression of CYP1B1 in this cell line. Tests performed on MCF-7 and MDA-MB-231 cancer cell lines showed that 5 µm of SFN significantly reduced mRNA and protein levels of CYP19 in MCF-7 cells, while increased mRNA and protein levels of CYP19 were observed in MDA-MB-231. At the same time, the protein level of CYP19 in non-tumorigenic MCF-10F cells was unchanged [90]. The data presented strongly indicate that the effect of SFN on the cytochrome activity with respect to chemopreventive activity of SFN needs further research.

#### 5.1.2. Phase II and Phase III of Xenobiotic Metabolism

Extensive literature suggests that a major component of the chemopreventive activity of SFN involves the induction of genes encoding phase II detoxification enzymes, a diverse family of enzymes that can metabolize and utilize a variety of reactive carcinogens, mutagens and other toxins. Such enzymes include NAD(P)H:quinone oxidoreductase (NQO1), GST, heme oxygenase-1 (HO-1), superoxide dismutase (SOD) and catalase (CAT), and they all contain antioxidant response elements (ARE) in the promoter regions, which in turn is a target for the nuclear transcription factor, erythroid 2 like 2 (Nrf2, NFE2L2) [91]. Under basal conditions, Nrf2 is bound to the Kelch-like ECH-associated protein 1 (Keap1) which targets Nrf2 for ubiquitination and proteasomal degradation. It is widely believed that electrophiles, such as SFN, disrupt Nrf2–Keap1 interactions, resulting in Nrf2 release and translocation to the nucleus [92,93]. According to the most recent theory, newly synthesized NRF2 is translocated directly into the nucleus, while the Keap1-mediated proteosomal degradation of Nrf2 is disrupted by stress factor. Hence, the de novo synthesized Nrf2 can subsequently accumulate in the nucleus by bypassing the ineffective Keap1 [94]. In the nucleus, Nrf2 binds to ARE sequences and thereby activates transcription of target phase II enzymes.

The literature data indicates that the effect of SFN on the phase II detoxification enzymes is selective. Yang et al. showed that 10 μM SFN produced an almost 3-fold induction of NQO1 activity in normal human breast cells MCF-10A [95]. Furthermore, Agyeman et al. showed that in the MCF-10A and MCF-12A cell lines, cytoprotective enzymes such as NQO1, GSTM1, SQSTM1(sequestosome 1) were up-regulated upon SFN treatment [96]. Conversely, Jiang et al. showed that 25 μm SFN inhibited the NQO1 activity to 79.4% of the control level in MDA-MD-231 cells, and GR (glutathione reductase) activity in MCF-7 and MDA-MB-231 cells (18.8% and 38.5%, respectively). Additionally, no statistically significant impact of SFN on GST and AR (aldehyde reductase) activity was observed [97]. This could suggest that SFN regulate Nrf2/ARE signaling pathway differently in normal and breast cancer cell lines, as was previously proposed by our team in relation to colon cells [98]. Indeed, Bose et al. demonstrated that a 2.5 μm dose of SFN did not alter the Nrf2 activity in MCF-7, MDA-MB-231 and MAT B III cells, while they detected enhanced Nrf2 activity in normal epithelial breast cells MCF-10A. Importantly, however, they detected high basal levels of nuclear Nrf2 activity in this three invasive cancer cell lines [99]. Now, Hu et al. showed that 1 μm and 5 μm doses of SFN in MCF-7 and MDA-MB-231 increased Nrf2 nuclear protein level. This result was associated with increased mRNA levels for some of the genes encoding detoxification enzymes, which are known to be Nrf2-regulated, as well as with increased protein levels of the following transcriptionally activated genes: NQO1, *γ*GCS (*γ*-glutamylcysteine synthetase) and GSTP1 [100]. The results are important, as high level of Nfr2 and NQO1 is associated with poor prognosis for breast cancer patients. Zhang et al. showed that compared to non-metastatic tumors, NQO1 showed higher expression in tumors with metastases. High Nrf2 expression strongly correlates with higher proliferation and migration of breast cancer cells, which predicts shorter survival time and higher recurrence rate in breast cancer patients [101]. Thus, a molecule which exhibits selectivity towards normal and cancer breast cells could find a potential application as a strategy to lower the Nrf2 level and improve current breast cancer therapies, or as could be proposed for SFN based on the literature data, as a strategy to increase the detoxification system of normal cells with no effect on cancer tissue, which in turn could mitigate the toxic effects of therapeutics.

To date, no data is available on the role of SFN on these III phase enzymes. The term Phase III xenobiotic metabolism refers to protein transporting systems, which are designed to maintain cellular homeostasis. This group includes P-glycoprotein (PGP) and Nrf-2-dependent multidrug resistance proteins (MRPs), which are responsible for moving xenobiotics outside the cellular membrane. In cancer cells, these proteins, similarly to proteins of phase II metabolism, are behind the enhanced cellular efflux of a wide variety of structurally distinct classes of chemotherapeutic agents and contribute to the multi-drug resistance (MDR) phenomenon, and thereby to the therapeutic failure and death in cancer treatment [102].

Only one report concerns the effect of GSTP-1 and MRP1 expression on the intracellular accumulation of SFN/SFN-SG and the nuclear level of Nrf2. By altering SFN intracellular level, MRP1 and GSTP1-1 affect ARE-dependent gene expression mediated by changes in the nuclear Nrf2 levels. The studies described suggest that the GST and MRP phenotype of a particular cell type or tissue may have a profound effect on the ultimate response to chemopreventive SFN [103].

To summarize, the results concerning the effect of SFN on the Nrf2-related detoxifying enzymes in normal and breast cancer cells show that in normal breast cancer cells, SFN increases enzymatic activity, which constitutes one of chemoprevention strategies and assists in maintaining the homeostasis in the body. At the same time in breast cancer cells a decrease is observed, that is also an advantageous effect, since an inhibition of the detoxification system (and in particular Nrf2 protein level) in cancer cells is a strategy to overcome a multi-drug resistance. It is widely acknowledged, that in cancer patients, a high levels of Nrf2 were recorded and that was a factor correlated with poorer prognosis [104,105,106]. If an induction of its activity by SFN is observed in cancer cells it should be recognized as unwanted, severe side-effect, which should exclude SFN from further study. Meantime, as discussed above, in case of SFN and breast tissue, at the level of in vitro study the selectivity was showed. The ability of SFN to decrease Nrf2 activity in breast cancer cells is beneficial from the point of view of cancer therapy, while its ability to increase Nrf2 activity in normal breast cells provides an useful and effective tool for cancer prevention.

#### 5.1.3. SFN Inhibits Growth of Breast Cancer Cells

Inhibition of tumor cell proliferation or the elimination of tumor cells represent another strategy of cancer chemoprevention, in particular in terms of secondary or tertiary prevention. Studies have shown that SFN inhibits the growth of breast cancer cells that represent different types of cancer. Table 4 presents the IC_50_ value (SFN concentration required to inhibit cell proliferation by 50%) of various breast cancer lines.

Most studies involved two cell lines: estrogen receptor positive MCF-7 and triple negative MDA-MB-231. In the studies of the MCF-7 versus MDA-MB-231 lines, the most pronounced differences in SFN sensitivity were observed after short incubation period of 24 h. On average, after 24 h incubation with SFN, the IC_50_ values for MCF-7 were lower compared to MDA-MB-231. The IC_50_ values were determined at 12.5 µm to 54 µm, and at 19.35 µm to 115.7 µm for MCF-7 and MDA-MB-231, respectively. After 48 h incubation with SFN, the IC_50_ values were determined at 7.5 µm to 27.9 µm, and at 8.3 µm to 16.04 µm for MCF-7 and MDA-MB-231, respectively. However, after 72 h of incubation with SFN, IC50 values indicate a similar sensitivity of cell lines to SFN, with IC_50_ values ranging from 6.73 µm to 33.8 µm for MCF-7 line, and from 7.26 µm to 31.5 µm for MDA-MB-231 line (Table 4) [107,108,109,110,111,112,113,114,115,116,117]. Longer incubation with SFN of 96 h results in the IC_50_ of 22 µM for MCF-7 and 46 µm for MDA-MB-231 [121].

The general pattern observed in the breast cancer lines was a micromolar range of IC_50_ and a decrease in IC_50_ values with longer exposure times to the SFN. It is striking that different studies report IC_50_ values that vary to a great degree. This is due to a number of factors. One issue is the selection of the test to determine the IC_50_. In this case, most tests were performed using the MTT method [108,109,110,112,113,114,116,117], while the other ones included trypan blue [107,111,121], sulforhodamine B [115] and crystal violet (CVS) [112] assays. These tests can yield different results, as was already widely discussed in the literature [122,123]. Another aspect pertains to the laboratory accuracy and precision. This has also been acknowledged and discussed in the scientific community. Among many other factors, the origin of a particular cell line, its identity and the method of propagation seem to play a significant role as the sources of discrepancies between various researches. This review produces yet another evidence that some effort should be made to standardize the cytotoxicity assessment [124].

The IC_50_ value for SFN was also determined for other breast cancer cell lines. Pledgie-Tracy et al. showed that after 48 h incubation of MDA-MB-468 and T47D cells with SFN, the IC_50_ was 8.1 µm and 9.5 µm, respectively [113]. Burnett et al. obtained similar results for SUM149 and SUM159 cell lines after 72 h incubation with SFN (7.5 µm and 7.8 µm, respectively) [118]. Kanematsu et al. showed that SFN directly inhibited growth of a human breast cancer cell line derived from a malignant effusion of a breast cancer patient, i.e., KPL-1 cell line. The IC_50_ values after 48 h and 72 h incubation were 19.1 µm and 17.8 µm, respectively (Table 4) [119].

Several studies were performed to identify the role of receptors in the cellular response to SFN. Pledgie-Tracy et al. have shown that 48 h incubation with 15 µm and 25 µm SFN down-regulated the protein expression of epidermal growth factor receptor (EGFR) and human epidermal growth factor-2 (HER-2) in MDA-MB-231, MDA-MB-468 and T47D cancer lines, while also down-regulating the protein expression of estrogen receptor (ER) in MCF-7 and T47D cells [113]. Pawlik et al. tested sensitivity to SFN of four breast cancer lines of distinct characteristics in terms of the status of pro-survival PI3K-AktmTOR-S6K1 pathway. In breast cancers, PI3K-AktmTOR-S6K1signaling pathway is often hyperactive due to overexpression of genes encoding growth factors or estrogen receptors, constitutive activation of PI3K or Akt and loss of phosphatase and tensin homolog (PTEN), a negative regulator of this pathway. The study included: MCF-7-an estrogen receptor (ER) positive cell line with low level of HER2 and EGFR1 receptors, MDA-MB-231-an estrogen-independent cell line with low level of ErbB2 and EGFR1 receptors, MDA-MB-468-cells lack the PTEN repressor and have high EGFR1 level, while SKBR-3 cells overproduce receptor kinase ErbB2. Regardless of their PI3K-AktmTOR-S6K1 status, the IC_50_ values ranged from 19 µm (for MCF-7) to 25 µm (for SKBR-3) after 24 h of treatment [109]. Similar results (IC_50_ values of approx. 20 µm) were also obtained by Azarenko et al., Lewinska et al. and Yang et al. for MCF-7, MDA-MB-231, SK-BR-3 and BT549 after 24 h incubation with SFN (Table 4) [108,111,117]. These results indicate that, despite different signaling at the level of receptors, SFN decreases survival of all tested cell lines to a similar degree. When taking these results into consideration together with previously demonstrated lack of substantial differences between growth inhibition of cells lines with different receptor status induced by SFN, it may be suggested that the mechanism of action of SFN in breast cancer cells is receptor-independent.

The data compiled in Table 4 indicates that in studies using both breast cancer cell lines and non-transformed breast lines, the activity of SFN is weaker in cancer non-transformed cells than in cancer transformed cells. IC_50_ for MCF-12A cell line after 48 h incubation with SFN was significantly higher (40.5 µm) than for breast cancer transformed cells, MCF-7 (27.9 µm) [115]. However, after prolonged incubation (72 h), the IC_50_ value for cancer non-transformed breast cells MCF-10 was slightly higher (12.4 µm) than the value obtained in cancer transformed breast cells MCF-7 and MDA-MB-231 (11.9 µm, 11.3 µm, respectively) (Table 4) [120]. In some studies, cells from other organs, such as dermal microvascular endothelium cells HMEC or CRL-1790, i.e., normal colon cells, were used as a model of normal cells. In case of HMEC line, after 24 h of incubation with SFN the IC_50_ index was almost four times higher (81.24 µm) than for cancer transformed breast lines MCF-7, MDA-MB-231 and SK-BR-3 [108]. The results obtained by Cierpiał et al. indicate that the selectivity of SFN activity on cancer breast cells and normal intestinal cells vary with the time of incubation. While the IC_50_ value obtained after 24 h was higher for breast cancer cells versus cancer non-transformed cells, after 48 h and 72 h, SFN induced an average two-fold growth inhibition of breast cancer cells MCF-7 and MDA-MB-231 compared to normal cells CRL-1790 [112]. Overall, these results indicate that at concentrations inhibiting the growth of tumor cells, SFN interferes with the functioning of normal (dividing) cells to a lesser extent. A side effect of oncological drugs involves their toxicity, resulting in damage to normal dividing tissues such as bone marrow, intestinal epithelium or epidermis, or cardiotoxicity through damaging endothelial cells. This selectivity of action towards breast cancer cells versus normal cells as above is beneficial.

Very interesting results were achieved with breast cancer cells treated with SFN combined with another compound. For example, Royston et al. studied the effect of SFN combined with Withaferin A (WA) (a withanolide isolated from a winter cherry prevalent in India) on MCF-7 and MDA-MB-231 cell lines. The combination of 5 µm SFN and 1 µm WA after a 72 h incubation was more effective than either of the compounds alone for both cancer cell lines. The decrease in cancer cell viability of up to 40% was observed, yet without significant effects observable for control MCF-10A cells [125]. Similarly, the combination of SFN (5 µm or 10 µm) with a chemotherapeutic agent gemcitabine (5 mm or 10 mm) had a synergistic effect and resulted in the inhibition of MCF-7 cell growth [110]. Furthermore, Milczarek et al. showed that the combination of SFN with 5-fluorouracil produced a significant reduction in the cell growth of the breast cancer MDA-MB-231 cell line, and similarly doxorubicin (DOX) + SFN was shown by Mielczarek et al. to have a synergistic effect on MCF-7 and MDA-MB-231 cells [126,127]. Also, the combination of SFN and 5-fluorouracil exhibited an antagonistic interaction which decreased the toxicity of the cytostatic toward Chinese Hamster fibroblast V79 cells. These results indicate the potential for using SFN as an adjuvant in cancer therapy to reduce cytostatic concentration as well as side effects. This was discussed in greater detail by Mielczarek in a comprehensive review on the interaction of SFN with cytostatics [128].

#### 5.1.4. SFN Induces Cancer Cell Cycle Arrest and Death

Cancer cells are characterized by increased growth caused by disturbances in cell cycle regulation and resistance to apoptosis, i.e., programmed cell death, which is designed to remove damaged cells that are harmful to the host (such as cancer cells). Last chemopreventive approach involves inducing residual cancer cell death. A cancer cell to be eliminated by a chemical agent may: become arrested at a specific cell cycle checkpoint, repair the damage and resume proliferation, or proceed directly to apoptosis or another type of programmed cell death, such as autophagy, or undergo permanent cell cycle arrest, which results in senescence or mitotic catastrophe, i.e., a type of abnormal mitosis [129].

Cell cycle regulation is mediated by cyclins, cyclin-dependent kinases (CDKs) and cyclin-dependent kinase inhibitors. The formation of cyclin complexes with CDK results in the cell entering the next phase of the cell cycle. The main inhibitor of CDK is p21 protein, whose expression is regulated by a number of signaling molecules, including p53. Tumor suppressor protein p53 is also the main regulator of the Bcl-2 family proteins, which also play an inhibitory role in apoptosis. There are two variants of apoptosis: intrinsic and extrinsic. The intrinsic pathway is mitochondrial-dependent and regulated by the Bcl-2 family, consisting of anti-apoptotic proteins such as Bcl-2, Bcl-x, while the pro-apoptotic proteins include Bcl-10, Bax, and Bak. Bcl-2 family proteins are activated when stimulated, cytochrome c is released from mitochondria, and caspase-9 is subsequently activated. The extrinsic pathway is induced when the death receptors on the cell surface are engaged by cognate ligands of the tumor necrosis factor (TNF) family. This in turn activates caspase-8 through adaptor proteins that include Fas-associated protein with death domain (FADD). As a result of activation of both pathways, the executioner caspases-3, -6 and -7 execute apoptotic cell death [130].

The majority of reviewed studies indicate that in case of breast cancer cells, SFN induces cell cycle arrest in the G_2_/M phase. Different SFN concentrations, however, resulted in different end-point effect of the cell cycle block (Table 5).

Lewinska et al. showed that 24 h incubation with 5 µm and 10 µm SFN caused the accumulation of the MCF-7 and MDA-MB-231 cells in the G_2_/M phase of the cell cycle, Table 5. It was demonstrated for the first time that this SFN activity is due to epigenetic changes, resulting in global DNA hypomethylation and changes in the microRNA profile. The cell cycle block was persistent and resulted in cellular senescence [108].

Jackson et al. showed that after incubation times of over 24 h, a higher dose of SFN of 15 µm induced G_2_/M cell cycle arrest in MCF-7 cells combined with mitotic arrest involving perturbation of tubulin polymerization and/or microtubule dynamics, which ultimately led to the mitotic catastrophe. Additionally, similar G_2_/M accumulation was observed in ZR-75, BT-20, and MDA-MB-231 breast cancer cells indicating no correlation with the ER status (Table 5) [131]. Treatment with the same dose of 15 µm SFN of F3II sarcomatoid mammary carcinoma cell line derived from a clonal subpopulation of a BALB/c mouse mammary adenocarcinoma also resulted in G_2_/M cell cycle arrest, which in part appeared to be also due to mitotic arrest prior to metaphase as a result of altered tubulin polymerization and/or dynamics [132].

After 72 h incubation of MDA-MB-231 cells with high 30 µM SFN, Kanematsu et al. observed S and G_2_/M cell cycle arrest (Table 5) associated with increased p21^WAFI^ and p27^KIP1^ levels and decreased cyclin A, cyclin B1 and CDC2 levels. Cells treated with SFN exhibited an increase in the cleavage of caspase-3 and the decrease in Bcl-2 level, which eventually induced apoptosis [116].

In the context of breast cancer apoptosis induced by SFN, the intrinsic signaling pathways seem to play a major role. However, Pledgie-Tracy et al. showed that the activation of apoptosis after a 72 h incubation with 5 µm, 10 µm and 25 µm SFN in triple negative therapy-resistant breast cancer MDA-MB-231 cells was initiated by the induction of Fas ligand, which results in the activation of caspase-8, caspase-3 and poly(ADP-ribose) polymerase (PARP). Now, an intrinsic, mitochondrial pathway was initiated in MDA-MB-468, MCF-7 and T47D cell lines. In these cells, Bcl-2 expression was decreased, and a release of cytochrome c from the mitochondria to the cytosol and activation of caspase-9 and caspase-3 was observed. This data suggests that incubation with SFN induced apoptosis via either the caspase-8 or caspase-9 pathways in human breast cancer lines, depending on cell line [113].

The intrinsic signaling cascade activation by SFN was described also by other researchers in respect of the changes in Bcl-2/Bax ratio. Pawlik et al. showed that 5 µm SFN after 96 h of incubation decreased Bcl-2 protein level by 30 to 50% while increasing Bax protein level by approx. 50% in MCF-7, Bt-474 and T47D cells [133]. Royston et al. showed that there was an induced Bax expression after 72 h treatment with 5 µm SFN of MCF-7 and MDA-MB-231, whereas Bcl-2 expression was inhibited [125]. Jackson et al. showed that a higher SFN concentration of 15 µm induced a reduction of the Bcl-2 and PARP protein levels in F3II sarcomatoid mammary carcinoma cell line derived from a clonal subpopulation of a murine adenocarcinoma [132]. Similarly, Hussain et al. showed that 25 µm SFN significantly reduced the expression of Bcl-2 in MCF-7 cells compared to untreated cells [110].

Sarkar et al. observed an increase in the expression of proapoptotic Bax and Bad proteins, with a concomitant decrease in anti-apoptotic Bcl-2 expression, with an intensification of the effect with increasing concentrations of SFN (1 to 20 µm). The study was performed on p53 wild type (wt) MCF-7 and p53 mutant MDA-MB-231 cells after 24 h incubation with SFN. This study also proposed that SFN induced apoptosis in breast cancer cells by down-regulating Heat Shock Proteins HSP27, HSP70, HSP90 and HSF1 (Heat shock factor 1) with subsequent up-regulation of p21 irrespective of p53 status [134].

Sarkar et al. have also shown that after 24 h SFN increased the expression of p53 and p21 in MCF-7, but in MDA-MB-231 (mutant p53) only the expression of p21 was increased, while the expression of dysfunctional p53 was decreased. These results indicate that in case of SFN, up-regulation of p21 can occur independently of p53. However, subsequent down-regulation of pro-apoptotic and anti-apoptotic proteins resulting in the release of cytochrome c and activation of caspases, particularly caspases-3 and -9, was more prominent in MCF-7 cells. The same study also showed that no increase in apoptosis was observed upon SFN incubation in non-transformed MCF-12F cells [134].

Conversely, Lewinska et al. have shown that pro-apoptotic activity of 20 µm SFN was slightly more evident in MDA-MB-231 than in MCF-7 and SK-BR-3 cells, possible due to decreased phospho-ERK1/2 levels in MDA-MB-231 cells, but not in MCF-7 and SK-BR-3 cells. Also, the same SFN concentration did not promote apoptosis in non-transformed dermal epithelial cells HMEC, which, taking into account the results obtained by Sarkar et al., may suggest that pro-apoptotic activity of SFN is specific to cancer cells [108,134].

In the same study, Lewinska has also shown that the SFN activity is concentration dependent. At high SFN concentrations, its cytotoxic activity (apoptosis and cytotoxic autophagy) was observed in breast cancer cells, while cytostatic autophagy and cell senescence were observed at lower concentrations [108]. Similarly, Yang at al. have shown for MDA-MB-231 triple negative cell line that only high SFN concentrations induced apoptosis. Although the distortion in Bax/Bcl2 ratio was observed both at low and high SFN concentrations, only the higher SFN dose (25 µm) was shown to induce apoptosis in TNBC cells [117]. It was also observed by Milczarek et al. that after 72 h incubation with a similar SFN concentration, apoptosis was induced in over 20% of the population of MDA-MB-231 cells [126]. These results are in line with the previously discussed SFN behavior as a hormetic compound which, at low concentrations, induces prosurvival effect while being cytotoxic and directing cells to the death pathway at high concentrations [135].

Autophagy has been recently recognized as the second type of programmed cell death. Autophagy is a process of protein and organelle degradation which maintains the quality of cellular components [136]. Autophagy is a two-fold mechanism: a small increase in autophagy level in cells grants protection against apoptosis, while a marked prolongation of autophagy induces cell death. Thus, when the cells develop resistance to apoptosis, autophagy may be a new alternative way to eliminate cancer cells.

Also, Kanematsu et al. showed that longer incubation time of 72 h with a higher SFN dose of 30 µm induced autophagy in MDA-MB-231 cells. They detected autophagosomes, autolysosomes and an increased level of LC3-II protein, which is a specific marker of autophagy associated with increased LC3 mRNA expression [116]. Pawlik et al. found that a higher SFN dose of 40 µm combined with a shorter incubation time of 6 h resulted in the formation of vacuoles in the following four breast cancer lines: MCF-7, MDA-MB-231, MDA-MB-468 and SKBR-3. However, the largest number of vacuoles was found in SKBR-3 (HER+), which could suggest that the intensity of autophagy induction may correlate with cell type or HER status [109]. Yang et al. demonstrated autophagy induction in breast cancer cells MDA-MB-231, MDA-MB-468 and BT549 after 24 h of incubation with 10 µm SFN [117].

Yang et al. also showed that the occurrence of autophagy in MDA-MB-231 cells following a 5 µm and 10 µm doses of SFN after a 24 h incubation was mediated by the suppression of histone deacetylase 6 (HDAC6) mRNA expression. This contributed to the increased acetylation and membrane translocation of phosphatase and tensin homolog (PTEN), eventually resulting in autophagy [117]. Histone deacetylase (HDAC) inhibitors are powerful epigenetic regulators, which induce cancer cell cycle arrest, differentiation and cell death [137,138]. HDAC inhibition affects an important mechanism of gene regulation. A deacetylation of histone proteins, allows DNA to wrap tightly, which in turn results in gene silencing. Moreover, HDAC regulates the acetylation status of various non-histone proteins including transcription factors and signaling molecules. It was proposed by Pledgie-Tracy that SFN at concentrations of 5 to 25 µm may inhibit total HDAC activity in MCF-7, T47D, MDA-MB-231 and MDA-MB-468 breast cancer lines, although no significant changes in global histone H3, H4, or tubulin acetylation were observed. Inhibition was most pronounced in TNBCs: MDA-MB-231 and MDA-MB-468 cell lines [113].

Importantly, SFN may also have a potentially undesirable effect. Pawlik et al. showed that the combination of 5 µM SFN with 0.5 µm 4-hydroxytamoxifen (used in breast cancer prevention) in the breast cancer lines MCF-7 and T47D induced protective autophagy, while combination of 5 µm SFN with 1 µm 4-hydroxytamoxifen in BT-474 cells resulted in lower cell survival rates, possibly due to high basal level of autophagosomes, according to the author [133]. Conversely, Milczarek et al. showed that autophagy may be an alternative effective type of cell death for the combination of 5-fluorouracil and SFN, which synergistically reduced cell growth of MDA-MB-231 cells by inducing autophagic cell death and premature senescence [126].

#### 5.1.5. SFN Inhibits Breast CSC

CSCs are a population of cells with capabilities of self-renewal and differentiation, which may lead not only to tumor metastasis or recurrence, but are responsible for tumor initiation and development. Hence, CSC elimination except of being the therapy target should be also considered as a target for chemoprevention at every level. In 2010 Li et al. first demonstrated the SFN ability to reduce the breast cancer stem/progenitor cell population in vitro. Low doses of SFN (0.5 µm, 1 µm and 5 µm) decreased aldehyde dehydrogenase-positive cell population in human breast cancer SUM159 cells and decreased the quantity of MCF-7 and SUM159 primary mammospheres by 45 to 75%, while also inducing an 8 to 125-fold reduction in the size thereof [114]. The downregulation of the Wnt/beta-catenin self-renewal pathway was suggested as a possible mechanism of SFN inhibitory effect on breast CSCs. The next study in this group has confirmed that 2.5 µm and 5 µm doses of SFN helped eliminate breast CSCs and inhibited sphere formation, while impairing ability to self-renew in SUM149 and SUM159 breast cancer cells [118]. Furthermore, it was shown that SFN inhibited NF-*κ*B p65 subunit translocation from cytoplasm into the nucleus, thereby suppressing the expression of cytokines and promoters of critical importance in the regulation of CSC growth.

SFN was also shown to reverse taxane-induced enrichment of the CSC population. Treatment of SUM149 and SUM 159 cells with docetaxel and paclitaxel alone doubled the number aldehyde dehydrogenase (ALDH)-positive cells, while 2.5 µm and 5.0 µm doses of SFN lead to a decrease in ALDH-positive cells by 40 to 50%. Moreover, the combination of SFN with either paclitaxel or docetaxel not only greatly enhances cytotoxic potency against bulk tumor cells, but also markedly suppresses the CSC population compared to paclitaxel or docetaxel alone [118].

The SFN properties described above shed a new light on its properties It can be assumed that this compound exhibit a very desired by now ability to potentially prevent initiation, development or a relapse of the breast cancer via elimination of CSCs. Moreover, combination of an anti-CSC agent (here SFN) with a chemotherapeutic agent may achieve much better clinical results in terms of long-term survival, which is particularly important when it comes to type 3 chemoprevention.

### 5.2. In Vivo Studies Demonstate the Efficacy of SFN

In vivo study is a critical and decisive stage for the assessment of the future perspectives of new drugs. There is also a number of in vivo evidence of SFN being involved in breast cancer, which takes it beyond the in vitro sphere.

The first reports on the chemopreventive activity of SFN in breast tissue concerned the inhibition of PAHs–DNA adduct formation which constitutes the initial stage of carcinogenesis. More recently, Kanematsu et al. showed that SFN prevents breast tumor formation. 25 mg/kg and 50 mg/kg of SFN were administered to mice subcutaneously injected with KPL-1 cells the day before. After 26 days, the tumor mass was observed to be reduced by 27% (after administration of 50 mg/kg SFN) and 41% (after administration of 25 mg/kg SFN) compared to control animals. SFN did not cause significantly lower body weight and did not body weight loss in the animals [119]. These results demonstrate the chemopreventive effect of SFN during tumor development, manifested in the deceleration thereof.

The ability of SFN to reduce the tumor size in mice was also shown in several studies. Jackson et al. conducted a study where BALB/c mice were subcutaneously injected with BALB/c murine mammary carcinoma cell line F3II, and 5 days later they were injected with 15 nmol SFN for 13 days. As a result, the tumor mass decreased by 60%, which indicates that SFN is capable of destroying tumor cells even in very small doses in vivo. What was important, no symptoms of toxicity were observed following SFN administration [132]. Yang et al. performed an experiment on mouse xenografts injected with triple negative breast cancer MDA-MB-231 cells. Five days after implantation, intraperitoneal injections of SFN at 50 mg/kg were performed once a day for four weeks. The tumor mass in the animals treated with SFN was significantly lower (~20%) compared to the control animals. During the treatment of SFN was observed not significant animals weight loss [117]. This result was also confirmed by Burnett et al. They performed a subcutaneous injection of TNBC SUM149 cells, while using the same SFN dose (50 mg/kg) for an extended period of time (50 days) via intraperitoneal administration. Their research showed that SFN reduced bulk tumor volume by 37.4% versus control with no change in animals body weight [118].

Li et al. showed that SFN also eliminated breast CSCs in vivo. SUM159 cells were injected to the mammary fat pads of 5-week-old female NOD/SCID mice. Two weeks after cell injection the mice were administered SFN at 50 mg/kg. After two weeks of treatment, tumors in SFN-treated mice were 50% the size of that in control animals, while the population of ALDH (+) cells (having the self-renewal ability) in the tumor was reduced by over 50%. During the experiment, no toxic effects were observed in animals. Li et al. also examined the growth of secondary tumors in NOD/SCID mice inoculated with primary tumor cells derived from primary xenografts. The cancer cells obtained from SFN-treated mice largely failed to produce any tumors in recipient mice up to 33 days after implantation, while control tumor cells yielded tumors as early as on Day 7. This demonstrates that SFN was able to eliminate breast CSCs in primary xenografts, thereby abrogating the re-growth of tumors in secondary mice [114]. In line with the tertiary chemoprevention approach, these results confirmed SFN beneficial properties discussed at the in vitro level. What is worth noticing, SFN did not significantly lower body mass of treated animals in all cited studies and no other signs of toxicity were observed following SFN administration [114,117,118,119,132].

Cornblatt et al. conducted an in vivo experiment to evaluate whether SFN has a direct chemopreventive effect at a molecular level in animal and human untransformed mammary tissue. In female Sprague–Dawley rats’ mammary gland, an almost 3-fold induction of NQO1 enzymatic activity and 4-fold elevation of HO-1 protein level after oral administration of 150 µmol SFN were reported. The peak post-administration SFN metabolites concentration in breast tissue was 18.8 pmol/mg tissue and in plasma was 60 µm at 1 h after dosing. In the same study, the authors showed at approximately 1.5 h before the breast resection the presence of SFN metabolites in human mammary gland after a single oral dose of a broccoli sprout preparation containing 200 μmol of SFN. The mean SFN metabolites concentration in the right and left breast tissue was 1.45 ± 1.12 and 2.00 ± 1.95 pmol/mg tissue, respectively and in plasma was 0.92 ± 0.72 µm. The authors also reported that NQO1 and HO-1 transcripts were detected in these biopsy samples, as well as NQO1 enzymatic activity without, however, any comparative measurements being performed to show the effect of SFN digestion. The short interval between ingestion of the broccoli sprout preparation and breast resection did not allow to evaluate the pharmacodynamic effect in breast tissue [139].

The chemopreventive activity of SFN was also reported in clinical trial in women. In 2015, Atwell et al. evaluated the effect of SFN on 54 women who consumed for at least two weeks either 224 mg GFN (BroccoMax^TM^) or placebo. Total SFN metabolites concentration in plasma post-intervention was 0.25 µM. The results indicate that SFN decreased peripheral blood mononuclear cell (PBMC) histone deacetylase (HDAC) activity. They also observed a significant decrease in Ki-67 levels, a cell proliferation marker, following SFN supplementation in benign carcinoma [140].

Oral doses of SFN used in presented studies in human subjects resulted in SFN metabolites concentration in plasma 0.25–0.92 µm and in breast tissues 1.45–2 pmol/mg, while in rats respectively 60 µm and 18.8 pmol/mg [99,139,140]. According to pharmacokinetic studies concentration of SFN metabolites after dose of 200 µmol broccoli sprout resulted in 0.943–2.27 µm in human plasma [82,141]. Oral bioavailability of SFN varies depending on the form of administered broccoli. Comparing the doses of SFN used in vitro and in vivo the received concentration of SFN metabolites was lower in human plasma than SFN concentration used in vitro, or in animal studies. This relation was also noticed by Yagishita et al. In this review on the relationships between formulation, bioavailability, and the doses of glucoraphanin and/or sulforaphane that have been used in pre-clinical (in vitro and in vivo) and clinical studies the authors strongly advice that the in vitro and in vivo studied doses should be attainable and tolerable in humans [142].

There are also reports indicating the usefulness of SFN combined with conventional chemotherapy. Phase II clinical study on safety of SFN and DOX administration in women suffering from breast cancer was started in 2019. SFN is tested as an adjuvant to attenuate DOX-induced cardiomyopathy, and possibly enhance the treatment effect of breast cancer. Previous studies in animal models already delivered evidence that SFN may attenuate the cardiac toxicity of DOX treatment. The results indicate that SFN at the dose of 4 mg/kg/day enhanced mitochondrial respiration in the hearts of 20 mg/kg DOX-treated female Sprague Dawley rats and reduced cardiac oxidative stress caused by DOX, as evidenced by the inhibition of lipid peroxidation, the activation of Nrf2 and associated antioxidant enzymes such as SOD, aldo-keto reductase 1 (AKR), aldehyde dehydrogenase (ALDH) and CAT. The enzymes activities were elevated above control levels in rats treated with SFN. In animals co-treated with SFN and DOX, activities of all enzymes were essentially restored to the levels present in control animals [99].

## 6. SFN Analogs

In the literature there is a variety of materials discussing the effect of SFN analogs and derivatives on breast cancer and non-transformed cells. It has been shown that even a small change in the SFN structure can cause significant difference in its biological activity. Table 6 compiles candidate structures for breast cancer chemoprevention.

Erucin ((4-methylthio) butyl ITC, ERN) is abundant in the rocket salad (*Eruca sativa* Mill.). ERN is a reduced analog of SFN and it is formed both by way of hydrolysis of the glucosinolate glucoerucin by the enzyme myrosinase and in vivo reduction of SF. Compared to SFN, erucin lacks the oxygen atom in the sulfinyl group (Table 6) [143]. In general, ERN and SFN have comparable effects. ERN was shown to decrease the viability of MDA-MB-231, SKBR-3 and T47D breast cancer cell lines. ERN induced cell cycle arrest in the G_2_/M phase, down-regulated the phosphorylation of S6 ribosomal protein in all tested breast cancer cell lines, and reduced HER2 receptor levels in SKBR-3 cells [145]. Pawlik et al. showed that after incubation of MCF-7, T47D and BT-474 cells with erucin, IC_50_ values were 9.7 µm, 7.6 µm and 19.7 µm, respectively, similar to SFN (5 µm, 6.6 µm and 15 µm). It was also shown that ERN (5 µm) in combination with 4-hydroxytamoxifen induced apoptosis of MCF-7 and T47D breast cancer cell lines and protective autophagy in MCF-7 and T47D cell lines, while contributing to lower survival in BT-474 cells. This effect is mediated by downregulation of anti-apoptotic proteins such as Bcl-2 and survivin and, as a result, by the induction of cell death, and in case of autophagy by the conversion of soluble LC3-I to the lipid-bound LC3-II form, which is an established autophagy marker. This analog also efficiently sensitized tamoxifen-resistant variants of MCF-7 and T47D cells to 4-hydroxytamoxifen [133]. Jackson et al. also showed that 100 µmol/L ERN significantly inhibited the polymerization rate, as well as the maximum polymerization, of purified tubulin proteins in MCF-7 cells similarly to SFN [131].

Sulforaphene (4-isothiocyanato-1-methylsulfinylbut-1-ene, SF) is a structural analog of SFN which differs only in the double bond in the alkyl chain (Table 6). It is formed during hydrolysis by myrosinase of glucoraphenin (4-methylsulfinyl-3-butenyl glucosinolate), which is an important component of radishes (*Raphanus sativus* L.), especially their seeds. SF efficiently decreased the viability of breast cancer cells SKBR-3 and MDA-MB-231, while normal breast cells (MCF-10A) were less sensitive. After 24 h incubation with SF, IC_50_ for SKBR-3 was 15 µm and 11 µm for MDA-MB-231, which was lower compared to SFN. SF at concentrations of 2.5 to 20 µm also induced cell cycle arrest in the G_2_/M phase and disturbed cytoskeletal organization and reduced clonogenic potential of SKBR-3 and MDA-MB-231 cancer cells. At the same concentrations, SF caused apoptosis in the concentration-dependent manner, which was associated with oxidative stress, mitochondrial dysfunction, increased Bax:Bcl2 ratio and ADRP levels in SKBR-3 and MDA-MB-231 cells [144].

Apart from the naturally occurring isothiocyanates, there are also several synthetic analogs, which exhibit interesting chemopreventive activity. The best known ones include Alyssin (isothiocyanate 5-methylsulfinyl-*n*-amyl) and ITC-2-oxohexyl (iso-thiocyanate-2-oxohexyl). Alyssin has the methylene chain extended with one -CH_2_ group in comparison to SFN, while ITC-2-oxohexyl was obtained as a result of replacing the sulfinyl group with carbonyl in SFN (Table 6). Alyssin and ITC-2 decrease the activity of enzymes CYP1A1 and CYP1A2 induced by benzo[a]pyrene in MCF-7 breast cancer cells. Alyssin directly reduced the catalytic activity of CYP1A1 in all concentrations used (0.5–2.5 µm) up to 75%, while ITC-2-oxohexyl caused linear CYP1A1 catalytic activity inhibition combined with an increase in the concentration (0.5–2.5 µm) up to 72% of initial activity. The effect occurred through direct inhibition of enzymatic activity and enzymatic protein depletion, which made it more efficient compared to SFN [146]. In another publication, Skupinska et al. studied the potency of these analogs in concentrations of 0.5, 1, and 2.5 µm to inhibit CYP1A1 and CYP1A2 enzymes induced by PAHs, dibenz[a,h]anthracene (DBA) and anthracene (ANT) in MCF-7 cell line. Allysin showed the strongest inhibitory properties for CYP1A1, whereas for CYP1A2 the inhibition pattern observed was similar to that of SFN. ITC-2-oxohexyl did not inhibit enzymatic activity of the CYP1A2, and the CYP1A1 was affected only by the PAH highest concentration [89]. This result suggests that elongation of alkyl chain (Alyssin) is a modification which can even enhance the chemopreventive properties of the ITCs, while the replacement of the sulfur atom with carbon in alkyl chain of ITC molecules reduces ITC-2-oxohexyl inhibitory potency. The replacement of sulfur atom with carbon may lead to alteration of charge localization in the ITC and modify the molecular activity.

The effect of replacement of the sulfur atom by selenium in the original isothiocyanate moiety was studied by Cierpiał et al. This study involved a synthesis of 8 new sulfides and 4 new sulfoxides. Apart from the replacement of the sulfur atom, they differed in terms of the length of methylene chain, presence of benzyl, and the number of substituents, mainly fluorine atoms. They generally had a significantly higher cytotoxicity against breast cancer cell lines than the original SFN, while being less toxic for human non-malignant CRL-1790 colon fibroblasts used as a model of a normal cell line. Among all of the compounds tested, 4-isoselenocyanato-1-butyl fluoroaryl (fluorobenzyl) sulfoxides, Table 6, proved to be the most efficient in breast cancer cell rate reduction. The most interesting compound is perhaps 4-isoselenocyanato-1-butyl 4′-fluoro-benzyl sulfoxide, since it has the highest selectivity index (SI = 12.6) between the triple-negative MDA-MB-231 breast cancer cell line and normal colon cells CRL-1970. Furthermore, its IC_50_ value after 72 h was twice as high in normal cells (34.22 µm for CRL-1790), and twice as low as that of SFN in cancer cells (3.47 µm for MCF-7; 2.72 µm for MDA-MD-231). The results obtained by Cierpiał indicate that the minimal changes made in the original SFN structure, such as the replacement of hydrogen atoms with fluorine atoms, and replacement of the isothiocyanate group with an isoselenocyanate moiety improved the compound’s ability to inhibit the growth of selected breast cancer cells (MCF-7, MDA-MB-231). The results also suggest that the increased cytotoxicity of the newly synthesized compounds may be due to a synergistic effect of both the organofluorine and isoselenocyanate moieties [112].

Although still little, there is some evidence in literature that the synthesis of a new SFN analog could yield a new active compound with beneficial properties in breast cancer chemoprevention. The literature review indicate that the synthetic analogs are more likely be a candidate for chemopreventive drug rather than natural ones. The most beneficial structural alterations include: the elongation of carbon chain and introduction of selenium and fluorine atoms into the molecule. However the above discussed studies were conducted at the in vitro level thus due to the lack of sufficient in vivo results it is too early at the moment to decide whether these analogs may be practically implemented as the therapeutics. Presently, SFN remains the most promising molecule and the closest future for breast cancer chemoprevention. There is no doubt, however that, more effort should be put in the conducting in vivo studies on the most promising analogs, i.e., alyssin or 4-isoselenocyanato-1-butyl 4′-fluoro-benzyl sulfoxide.

## 7. Conclusions

Despite a reduction in mortality observed in recent years, breast cancer is the main cause of cancer-related deaths among women, and therefore remains a critical problem not of health alone, but also in socio-economic terms. Therefore, chemoprevention, including pharmacological chemoprevention, is an important aspect that should be considered to a greater extent. Currently, there are two drugs on the market used in tertiary chemoprevention of breast cancer, and there are several more in clinical trials. These are mainly already registered drugs for which new indications are sought.

SFN, a compound of natural origin, whose detoxifying and therefore potentially chemopreventive properties were discovered 30 years ago, increasingly features in various studies (including clinical trials) regarding its use in breast cancer therapy. Many centres are also endeavouring to identify its active, synthetic analogues. In various forms of often dubious quality, SFN is available on the market as a food supplement, so that the scientific, medical and pharmaceutical community in particular should intensify the efforts to determine its therapeutic properties. Nowadays, there are many dietary supplements available, highly popular among those who seek protection from cancer, but also–producing ambivalent effects–those during remission of the disease or after recovery intending to prevent relapses, metastases or secondary cancer.

The material compiled in this review allows us to conclude that the properties of SFN are promising and desirable in the context of its use as a drug in the prevention of breast cancer at every stage, also due to its low toxicity in normal tissue and additionally its ability to maintain normal homeostatic functions in the body. Currently ongoing clinical trials, when completed, are going to bring us closer to understanding whether it will be possible to introduce SFN to the drug market. The application of nanotechnology to elaborate the effective SFN carrier would be advantageous since it could enhance SFN stability and thus its efficacy. Such formulation, in our opinion, could be a future for breast cancer chemoprevention. As the closing remark it is worth mentioning that, it would be advantageous to step up efforts to determine the usability of selected SFN analogs (in particular those with introduced selenium and fluorine atoms) for use in chemoprevention, which would increase the range of pharmaceuticals that can be used either alone or in combination with existing drugs.

## Figures and Tables

**Table 1:** Chemical structures of new compounds in breast cancer prevention.

Name of Compound	Chemical Structure	References
Anastrozole	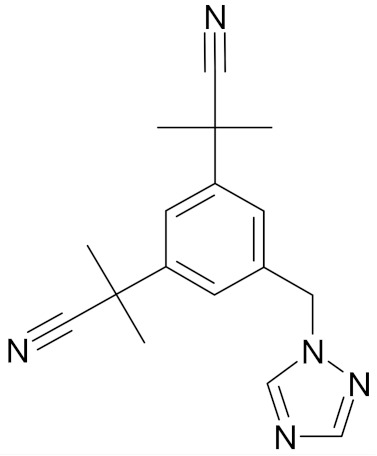	[47]
Exemestane	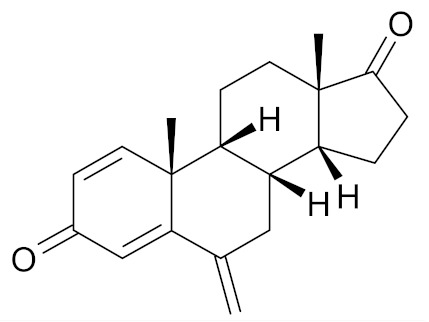	[48]
Lasofoxifene	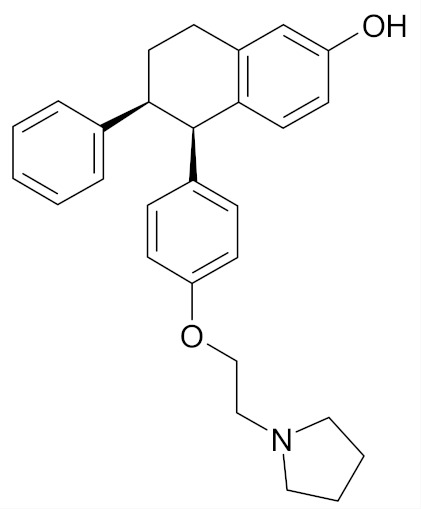	[49]
Pasireotide	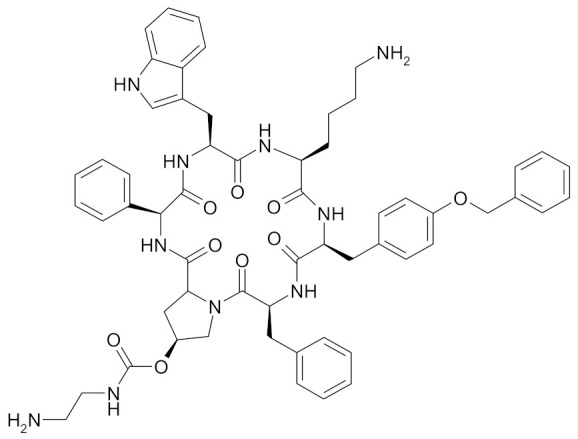	[50]
Arzoxifene	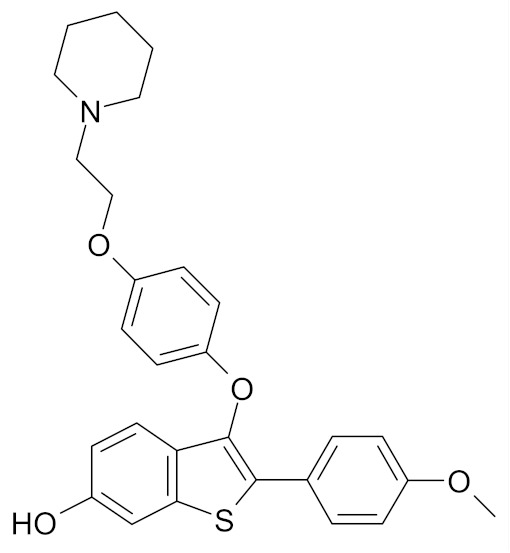	[51]
Lovastatin	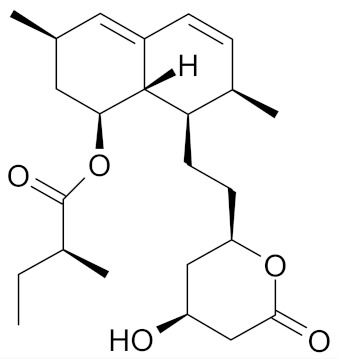	[52]
Alendronate	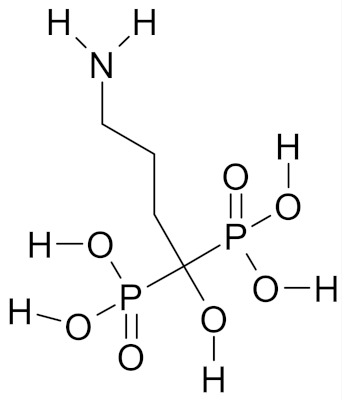	[53]
Letrozole	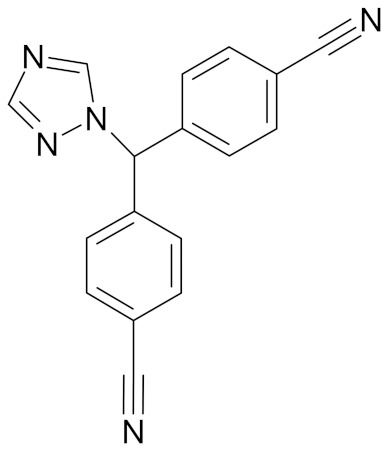	[54]
Deslorelin	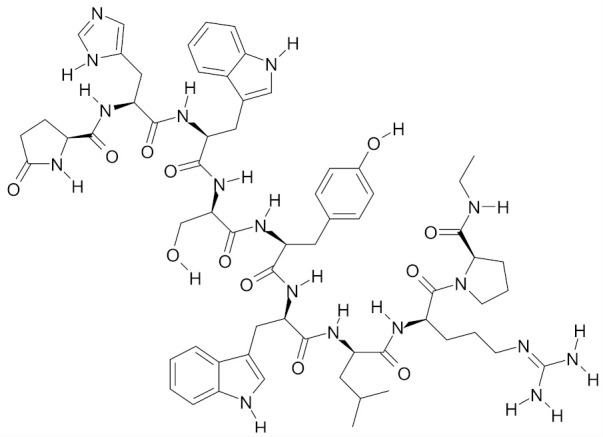	[55]
Sulindac	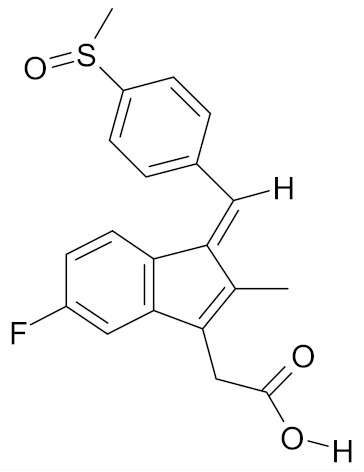	[56]
Metformin hydrochloride	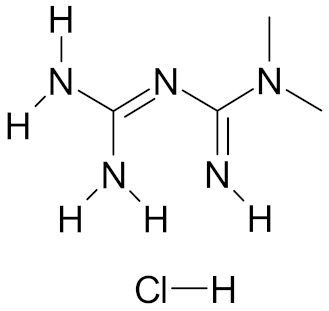	[57]

**Table 2:** Chemical structures of natural compounds in breast cancer prevention.

Name of Compound	Chemical Structure	References
Curcumin	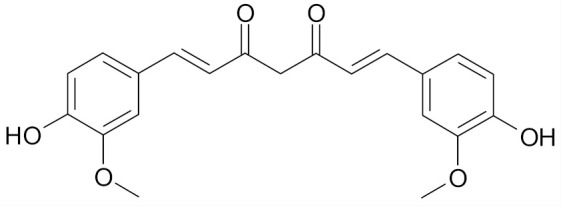	[66]
Genistein	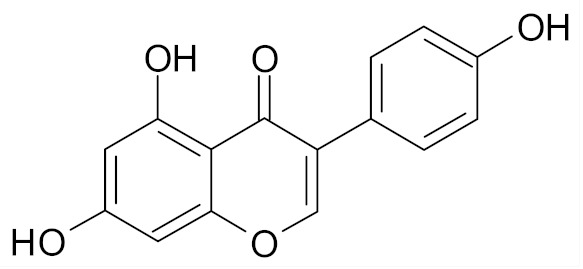	[67]
Resveratrol	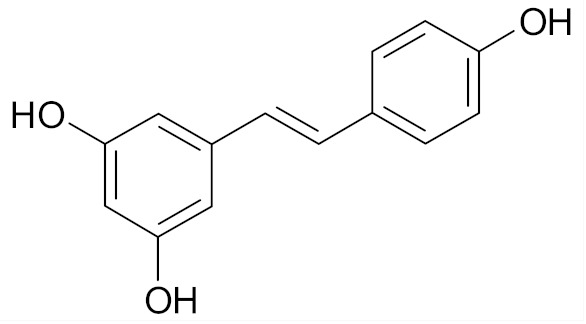	[68]
Epigallocatechin gallate	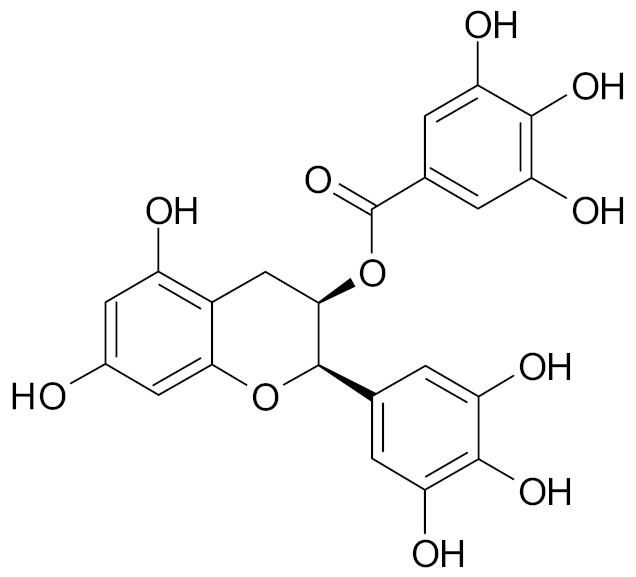	[69]

**Table 3:** Molecular data of breast cancer cell lines in comparison to normal breast cell lines.

	Receptor Status			Subtype	
Cell Lines	Estrogen Receptor (ER)	Progesterone Receptor (PR)	Human Epithelial Receptor (HER2)	Histologic	Molecular
MCF-7	+	+	-	invasive ductal carcinoma	Luminal A
MDA-MB-231	-	-	-	adenocarcinoma	Triple negative B
MDA-MB-468	-	-	-	adenocarcinoma	Triple negative A
BT549	-	-	-	invasive ductal carcinoma	Triple negative B
SUM149	-	-	-	inflammatory ductal carcinoma	Triple negative B
SUM159	-	-	-	anaplastic carcinoma	Triple negative B
BT-20	-	-	-	invasive ductal carcinoma	Triple negative A
SK-BR-3	-	-	+	adenocarcinoma	HER2 positive
T47D	+	+	-	invasive ductal carcinoma	Luminal A
ZR-75-1	+	-	-	invasive ductal carcinoma	Luminal A
MCF-12A	-	-	-	non-tumorigenic fibrocystic breast epithelium	basal
MCF-10A/F	-	-	-	non-tumorigenic fibrocystic breast epithelium	basal

**Table 4:** IC_50_ values for breast cancer and normal cell lines after 24 h, 48 h and 72 h of incubation with SFN.

Cell Line	24 h	48 h	72 h
MCF-7	12.5 µm [107] 14.05 µm [108] 19 µm [109] 25 µm [110] 26 µm [111] 38.92 µm [112] 54 µm [112]	7.5 µm [107] 9.2 µm [113] 12.38 µm [112] 13 µm [111] 16 µm [114] 20 µm [110] 20.06 µm [112] 27.9 µm [115]	6.73 µm [112] 9 µm [111] 10.15 µm [112] 33.8 µm [116]
		MCF-7/ADR 13.7 µm [115]	
MDA-MB-231	19.35 µm [108] 21 µm [109] 21.76 µm [117] 27.40 µm [112] 115.7 µm [112]	8.3 µm [113] 9.6 µm [112] 16.04 µm [112]	7.26 µm [112] 11.55 µm [112] 31.5 µm [116]
MDA-MB-468	20 µm [109] 21.93 µm [117]	8.1 µm [113]	-
SK-BR-3	16.64 µm [108] 25 µm [109]	-	-
SUM159	-	10 µm [114]	7.8 µm [118]
SUM149	-	-	7.5 µm [118]
BT549	20.47 µm [117]	-	-
T47D KPL-1	- -	9.5 µm [113] 19.1 µm [119]	- 17.8 µm [119]
MCF-12A	-	40.5 µm [115]	-
MCF-10A	-	-	12.4 µm [120]

**Table 5:** Phase of cell cycle arrest induced by SFN in breast cancer cell lines.

Phase of Cell Cycle Arrest	Time of Incubation with SFN	Cell Line	SFN Concentration
G_0_/G_1_	24 h	SK-BR-3	5 µm [108]
			10 µm [108]
G_2_/M	24 h	MCF-7	5 µm [108,111]
			10 µm [108]
			15 µm [111,113,131]
			25 µm [111]
	24 h	MDA-MB-231	5 µm [108]
			10 µm [108]
			15 µm [113,131]
	24 h	ZR-75	15 µm [131]
	24 h	BT-20	15 µm [131]
	72 h	MDA-MB-231	30 µm [116]
S	72 h	MDA-MB-231	30 µm [116]

**Table 6:** Chemical structures of SFN analogs.

Name of Compound	Chemical Structure	Reference
1-isothiocyanato-4-methylsulfinylbutane (sulforaphane, SFN)	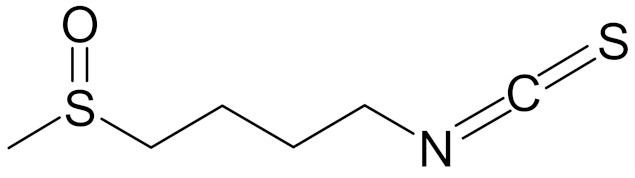	[89]
erucin ((4-methylthio)butyl ITC, ERN)	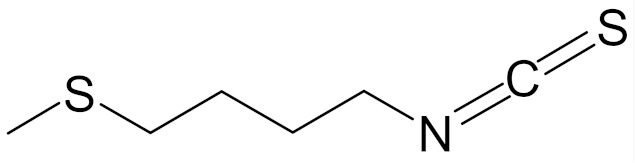	[143]
sulforaphene (4-isothiocyanato-1-methylsulfinylbut-1-ene; SF)	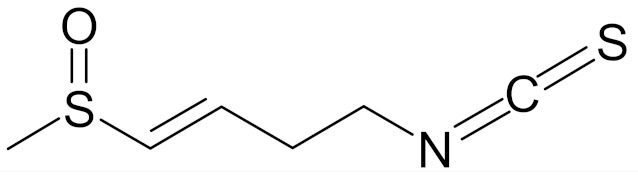	[144]
allysin (isothiocyanate 5-methylsulfinyl-*n*-amyl)	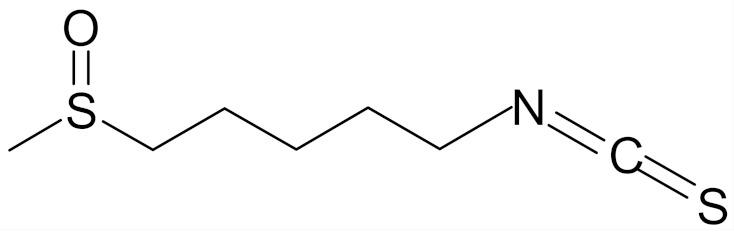	[89]
ITC-2-oxohexyl (isothiocyanate 2-oxoheksyl)	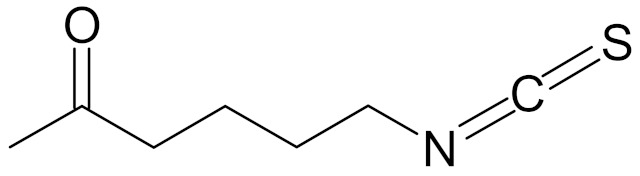	[89]
4-isoselenocyanato-1-butyl 4′-fluorobenzyl sulfoxide	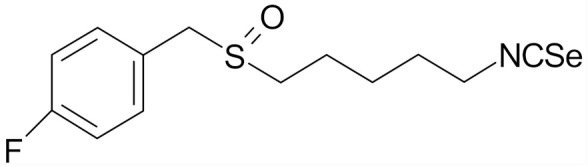	[112]
4-isoselenocyanato-1-butyl 3′,5′-bis-(trifluoromethyl)phenyl sulfoxide	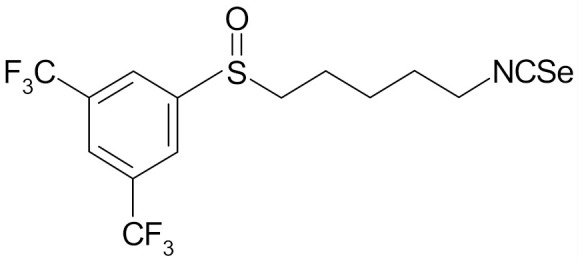	[112]
4′-(trifluoromethyl)-2′,3′,5′,6′-pentafluorophenyl-4-isothiocyanato-1-butyl sulfoxide	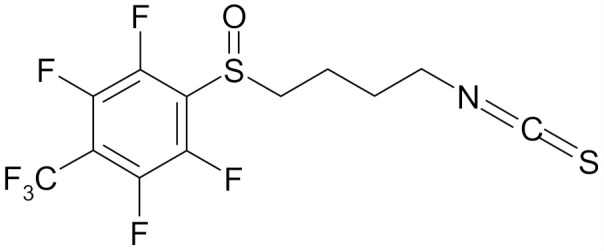	[113]
4′-(trifluoromethyl)-2′,3′,5′,6′-pentafluorophenyl-5-isothiocyanato-1-pentyl sulfoxide	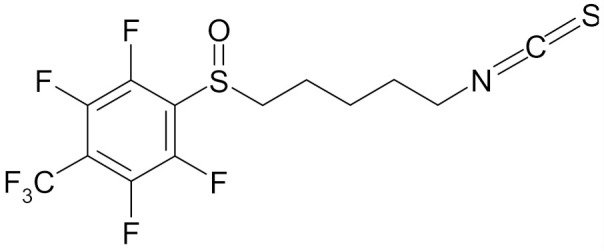	[113]
